# Supplementary material for: In vitro and in silico investigation of effects of antimicrobial peptides from Solanaceae plants against rice sheath blight pathogen Rhizoctinia solani
Source: PLoS One. 2024 Jun 13;19(6):e0302440. doi: 10.1371/journal.pone.0302440 (PMC11175423; doi:10.1371/journal.pone.0302440)
Supplement: Attachment — Submitted filename: Comments_Responses-R1-TIedited.docx [file pone.0302440.s001.docx]

**Responses to the reviewers' comments**

**Reviewer #1:**

**Reviwer’s comment:** The authors of a study have suggested that peptides derived from Solanaceous plants like Solanum tuberosum and Capsicum annum have antifungal properties against R. solani, a fungal pathogen. They have also performed molecular docking studies of these peptides with pathogenicity-related proteins of R. solani. The study did not present any new scientific hypothesis or concept. Therefore, my comments are:

There are some suggestions for improvement. Firstly, the abstract needs to be shortened to only present the silent findings of the research outcome.

**Authors’ response:** Thank you for the critical comment and valuable suggestion for the improvement of the manuscript. We substantially revised the manuscript and focused the novelty and perspective of our findings. The abstract is also shortened as suggested.

**Reviwer’s comment:** Secondly, performing q-PCR of blight resistance and sensitivity-related genes from treated and non-treated samples with peptides would validate the findings.

**Authors’ response:** We agree with the reviewer that q-PCR of blight resistance and sensitivity related genes from treated and untreated samples with peptides would validate our in vitro and insilico research findings. However, this approach was not the scope of the current study and it takes long time and resources. We are now planning to include this important idea in our new project as a follow up this study.

**Reviwer’s comment:** In the material method section, it is suggested to provide the full genus name for the first time appearing in the manuscript for C. annum and S. tuberosum.

**Authors’ response:** The full genus names are included for the first time appearing of C. annum and S. tuberosum, in the revised manuscript. Thank you so much for the comment.

**Reviwer’s comment:** Additionally, it is unclear if the author quantified the level of peptide and identified any.

**Authors’ response:** Thank you very much for your comment. Regrettably, our current laboratory capabilities and expertise in isolating peptides from plant extracts are very limited. Consequently, we have not undertaken quantification or characterization of antimicrobial peptide levels in peptide-rich fractions of C. annum and S. tuberosum leaves. We acknowledge the importance of this aspect and hope to explore collaborations or acquire necessary resources to address this in future studies. Your understanding of these constraints is greatly appreciated.

**Reviwer’s comment:** The extraction buffer used for peptide extraction should be mentioned too.

**Authors’ response:** Thank you very much for your comment. The optimized method reviewed by Barashkova & Rogozhin, 2020 and the method described by Astafieva et al., 2012 and Taveira et al., 2014 were adopted. This method does not require the use of a buffer.

**Reviwer’s comment:** Barashkova and Rogozhin, 2020 is a review article, so it should be clarified if the author made any modifications or standardized the protocol.

**Authors’ response:** Thank you very much for your valuable comment. We have adopted the protocol from the cited literature in this review (Astafieva et al., 2012; Taveira et al., 2014). Further, these references are cited in the methods section of the revised manuscript.

**Reviwer’s comment:** Moreover, it is unclear how the author deduced that the extract contains peptides that were used for docking.

**Authors’ response:** Thank you very much for your valuable comment. We deduced the presence of the peptides in peptide-rich extracts from C. annum and S. tuberosum leaves from the literature Afroz et al., 2020; Dev & Venu, 2016; Maracahipes et al., 2019; Moulin et al., 2014.

**Reviwer’s comment:** Lastly, the author needs to justify why they have selected prp Histone demethylase.

**Authors’ response:** Thank you very much for your valuable comment. Histone demethylase has been suggested as a target protein affecting biological processes such as pathogenicity, virulence, vegetative growth, and morphogenesis in *R. solani (*Prabhukarthikeyan et al., 2022). This compelled us to undertake a study on Histone demethylase.

**Reviwer’s comment:** The paper needs to be reorganized and proofread before submission.

Authors’ response: We agree with the valuable comment of the reviewer and hence substantially revised and reorganized the manuscript for coherence and clarity of the novelty and perspective of the findings. The language of the manuscript is checked thoroughly.

**Reviewer #2:**

**Reviwer’s comment:** The present article entitled "Antimicrobial Peptides from Solanaceae Plants: Analyzing Their Effect on Phytopathogenic Fungus Rhizoctonia solani" based on a good theme and authors have briefly discussed all the point. The presenation of the article is also fine.

**Authors’ response:** We sincerely appreciate the reviewer for this encouraging comment on our manuscript.

**Reviwer’s comment:** How ever I have only suggestion to improve the English of the article

**Authors’ response:** The manuscript was thoroughly proofread and substantially revised, including improving the English usage. Thank you very much for your valuable comment.
