# Supplementary material for: In vitro and in silico investigation of effects of antimicrobial peptides from Solanaceae plants against rice sheath blight pathogen Rhizoctinia solani
Source: PLoS One. 2024 Jun 13;19(6):e0302440. doi: 10.1371/journal.pone.0302440 (PMC11175423; doi:10.1371/journal.pone.0302440)
Supplement: Attachment — Submitted filename: Responses to the reviwers-R1.docx [file pone.0302440.s002.docx]

**Responses to the reviewers’ comments**

Reviewers' comments:

**Reviewer #1**

Reviewer #1: Authors have resolved the all my concern related to MS. Therefore, my decision to accept the ms for publication.

Response: Thank you for this encouraging decision.

**Reviewer #3**

Reviewer #3: The manuscript is of wrathful consideration for plant scientists. This is based upon effective study Antimicrobial Peptides from Solanaceae Plants: Analyzing, Their Effect on Phytopathogenic Fungus Rhizoctonia solan in that region. The write-up of whole manuscript is up to mark. English quality is acceptable but minor revision needed.

Response: Thank you. We revised the English usage, making the manuscript worthy.

However, there are some suggestions that need to be incorporated before final acceptance as elaborated below:

- Title of the manuscript should be changed.

Response: The title is revised as “In-vitro and In-silico Investigation of Effects of Antimicrobial Peptides from Solanaceae Plants on Rice Sheath Blight Pathogen”.

- The Abstract needs few details about materials of the experiments.

Response: This valid observation has been addressed in the abstract section of the MS as suggested.

- Please note the opening paragraph of the introduction could provide stronger context to the paper, and, similarly, the findings at the end could potentially be richer.

Response: The opening paragraph has already captured the context of the paper as it states the damaging effect of *R. solani* and possible yield loss due to the sheath blight pathogen infection. This explains the focus of the research for unveiling a safe method of the *R. solani* control.

- At the end of Introduction section, there should be clear hypothesis and objectives of the designed study.

Response: Thank you. The clear hypothesis has been incorporated at the end of the introduction section.

- Statistical designs and data analysis confusing. It needs to present in a proper way

Response: Statistical design has been revised and explained in a separate section 2.13 of the MS.

- Results presentations should be revised profoundly.

Response: We appreciate you for this encouraging comment. But the results were expressed in the best way it should be presented. This is evident when the figures and tables are observed.

- I suggest that the author should provide more justification for your study (specifically, you should expand upon the knowledge gap in the abstract, introduction, and all other sections being filled) which should be improved upon before Acceptance.

Response: the justification of the study, particularly in the introduction section, has been provided.

- The discussion need to revised and need to make it more focused based on results.

Response: Thank you. The discussion was rigorously based on the focus and results obtained from the study. These have been addressed from the beginning of the MS preparation.

**Reviewer #4**

Reviewer #4: (No Response)

**Reviewer #5**

Reviewer #5: Reviewer’s comment

The manuscript is written on potential antimicrobial peptides for the control of Rhizoctonia solani, causing rice sheath blight. Corrections are suggested below.

In abstract: A statement need to be included the confirmation of presence of peptides in the extracts.

Response: Thank you. An optimized method of AMP extraction was used as an extraction procedure in this study, according to these authors (Astafieva et al., 2012; Barashkova & Rogozhin, 2020; Taveira et al., 2014). Therefore, this requires no further confirmation of the AMPs extracted due to the notion that the extract obtained using this method contains AMPs. The plants used as a source of the AMPs were confirmed to possess these peptides, as established by Afroz et al. (2020).

In materials and method, et al., is written in italics. Should be same throughout the manuscript.

Response: All ‘et al’ in the updated MS have been italicized as suggested.

The field details (name, district, latitude, longitude etc.) under source of inoculum should be mentioned. Space should be given between 5 mm. 2.3 and 2.4 should be merged. Hour should be written as h. There should be space between ° and C.

Response: Field details have been provided in the MS. 2.3 and 2.4 were merged. All issues raised here have been addressed.

How do you know the extract is peptide rich? Include suitable references indicating peptide extraction protocol. Otherwise, characterization needs to be done.

Response: The plants used as a source of the AMPs were confirmed to possess these peptides as established by Afroz et al. (2020).

In-vitro should be in italics.

Response: Correction is made in the revised manuscript.

2.11 Once Rhizoctonia solani written in abstract, no need to mention complete name, only write R. solani should be written throughout the manuscript.

Response: The correction is done in the revised manuscript as suggested.

What are the names of the peptides present in the peptide rich extract? How do you prepare their structures? Mention the names and structures if someone has already been identified them previously from the plants taken.

Response: The structures of AMP from the *Solanaceae* plants were predicted from the PEP-FOLD3 server (Lamiable et al., 2016; Shen et al., 2014).

2.12. Molecular dynamics studies and MM-PBSA calculations section is very elaborative. It should be written concisely. Use references and avoid detail process.

Response: We agree with the concerns the reviewer raised. Wherever needed, the content is made concise. However, we feel that the details, whichever are kept in the revised manuscript, will give a deep understanding of the methodology and enhance the repeatability of the method.

For statistical analysis, atleast five test concentrations should be taken along with positive and negative control with three or more replications.

Response: This was what exactly was done during the analysis phase of the data obtained.

What was the positive control. Please mention the name and data in the table 1 and 2.

Response: DMSO was used as a negative control throughout the bioassay, while Azoxystrobin, a broad-spectrum systemic fungicide, was used as a positive control. Tables 1 and 2 were inserted in the positive control.

Table 4. Mention the source (reference from where the sequences of the peptides retrieved).

Response: This was fully mentioned in the “Protein-Peptide Docking Analysis” section of the manuscript.

The names of enzymes in Table 4 should be properly written. Write the unit of binding energy (kcal).

Response: We appreciate you for the kind observation. These details have been provided in the Table in the revised manuscript.
